# Nestin+ Peyer's patch resident MSCs enhance healing of inflammatory bowel disease through IL‐22‐mediated intestinal epithelial repair

**DOI:** 10.1111/cpr.13363

**Published:** 2022-11-20

**Authors:** Jieying Chen, Jing Huang, Jiahao Shi, Minrong Li, Erming Zhao, Gang Li, Xiaoyong Chen, Tao Wang, Qiaojia Li, Weiqiang Li, Jianping Ma, Wenzhe Mao, Rui Fang, Jiang Hao, Weijun Huang, Andy Peng Xiang, Xiaoran Zhang

**Affiliations:** ^1^ Center for Stem Cell Biology and Tissue Engineering, Key Laboratory for Stem Cells and Tissue Engineering, Ministry of Education Sun Yat‐sen University Guangzhou Guangdong China; ^2^ National‐Local Joint Engineering Research Center for Stem Cells and Regenerative Medicine, Zhongshan School of Medicine Sun Yat‐Sen University Guangzhou China; ^3^ Department of Medical Ultrasonic the Third Affiliated Hospital of Sun Yat‐sen University Guangzhou China; ^4^ Shenzhen Qianhai Shekou Free Trade Zone Hospital Shenzhen China

## Abstract

Inflammatory bowel disease (IBD) is a chronic condition characterized by gastrointestinal tract inflammation and still lacks satisfactory treatments. Mesenchymal stromal cells (MSCs) show promising potential for treating IBD, but their therapeutic efficacy varies depending on the tissue of origin. We aim to investigate whether intestine Peyer's patch (PP)‐derived MSCs have superior immunomodulatory effects on T cells and better therapeutic effects on IBD compared with bone marrow‐derived MSCs. We isolated PPs‐derived Nestin+ MSCs (MSCs^PP^) and bone marrow‐derived Nestin+ MSCs (MSCs^BM^) from Nestin‐GFP transgenic mice to explore their curative effects on murine IBD model. Moreover, we tested the effects of IL‐22 knockdown and IL‐22 overexpression on the therapeutic efficacy of MSCs^PP^ and MSCs^BM^ in murine IBD, respectively. We demonstrated that Nestin+ cells derived from murine PPs exhibit MSC‐like biological characteristics. Compared with MSCs^BM^, MSCs^PP^ possess enhanced immunoregulatory ability to suppress T cell proliferation and inflammatory cytokine production. Moreover, we observed that MSCs^PP^ exhibited greater therapeutic efficacy than MSCs^BM^ in murine IBD models. Interestingly, IL‐22, which was highly expressed in MSCs^PP^, could alleviate the severity of the intestinal inflammation, while knockdown IL‐22 of MSCs^PP^ remarkably weakened the therapeutic effects. More importantly, IL‐22 overexpressing MSCs^BM^ could significantly improve the symptoms of murine IBD models. This study systemically demonstrated that murine MSCs^PP^ have a prominent advantage in murine IBD treatment, partly through IL‐22.

## Graphical abstract

We isolated bone marrow‐derived Nestin+ cells (MSCs^BM^) and Peyer's patches‐derived Nestin+ cells (MSCs^PP^) with Nestin‐GFP transgenic mice. To systematically explore their properties, we analysed their proliferation abilities, differentiative potentials, especially immunoregulatory capabilities, and verified the feature using an inflammatory bowel disease (IBD) murine model. The results indicated that MSCs^PP^ exhibited far more superior efficacy than MSCs^BM^ in the treatment of IBD, mainly due to their high expression of IL‐22.

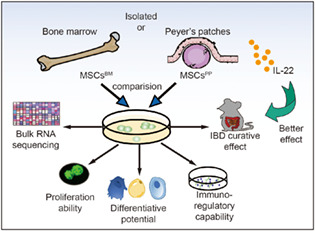

## 1 INTRODUCTION

Inflammatory bowel disease (IBD), including Crohn's disease (CD) and ulcerative colitis (UC), involves multifactorial inflammatory dysregulation associated with disordered relationships between the immune system and gastrointestinal tract environment.^1^, ^2^ Millions of incidences are recorded worldwide each year.^3^, ^4^ Patients with IBD mainly present with abdominal pain and diarrhoea and tend to experience complications such intestinal obstruction and fistulae. Nowadays, corticosteroids still are the mainstay of conventional therapy of IBD patients.^5^ However, when this strategy fails, patients are often subjected to intestinal resection to remove the affected gut.^6^, ^7^ In turn, surgery can be associated with inevitable complications, which lead to loss of function within the digestive system.^8^, ^9^ Therefore, developing new approaches for IBD is urgently needed.

Mesenchymal stromal cells are multipotent nonhematopoietic cells that distribute in most of the organs, and are characterized as multilineage differentiation, paracrine effects, and immunomodulatory activity.^10^ Preclinical and clinical data from animal models and humans have demonstrated that MSCs therapy is safe for treating IBD.^11^, ^12^, ^13^, ^14^ However, significant heterogeneity exists in terms of therapeutic efficacy. Molendijk et al. noted that the optimal dose of bone marrow‐derived MSCs failed to maintain long‐term efficacy of improvement.^15^ Similarly, Zhang et al. reported that in a study on umbilical cord‐derived MSCs, no patients achieved clinical remission after 12 months.^16^ While adipose‐derived MSCs are the most widely applied MSCs in IBD treatment, they still have exhibited inconsistent therapeutic effects.^17^, ^18^, ^19^, ^20^ All these discrepant clinical results indicated that various tissue‐derived MSCs may cause inconsistent treatment effects. A major hypothesis in the field of cell‐based therapy suggests that cell types derived from different tissues may have additive effects on tissue repair, probably because tissue‐resident MSCs are usually localized in a specific tissue microenvironment where they are thought to support continuous tissue homeostasis.^21^ However, the optimal source of MSCs and the underlying molecular mechanism of MSCs in attenuating IBD remain largely unknown. Therefore, we sought to identify the best source of MSCs for use in IBD therapy.

Secondary lymphoid organ Peyer's patches (PPs) provide the infrastructure for mucosal immune response maintenance and gut homeostasis, and their stromal subsets reside in specific anatomical locations in PPs and carry out distinct regulatory functions.^22^, ^23^, ^24^, ^25^ Hence, the interesting question of whether intestinal PP‐resident MSCs display distinct advantages over nonresident MSCs in the therapy of IBD has been proposed. Moreover, because the precise characteristics and functions of MSCs derived from PPs are still poorly understood. Therefore, we isolated MSCs from murine intestinal PPs to study their properties.

Nestin is a class VI intermediate filament protein that was originally described in neural stem and progenitor cells during embryonic development.^26^ Nestin is also expressed in MSCs of various tissues, including bone marrow, kidney, testis and heart,^21^, ^27^, ^28^, ^29^ suggesting that it can be used as a specific marker for isolating tissue‐resident MSCs. In this study, we found that Nestin can be used as a marker for identifying murine PP‐resident MSCs and then demonstrated that PP‐derived Nestin+ MSCs (MSCs^PP^) were more effective than bone marrow‐derived Nestin+ MSCs (MSCs^BM^) in treating murine inflammatory bowel disease, which was realized partially through interleukin 22 (IL‐22)‐mediated regulation of mucosal homeostasis.

## 2 METHODS

### 2.1 Mice

C57BL/6 wild‐type mice were purchased from the Animal Center at the Medical Laboratory of Guangdong Province, China. Homozygous Nestin (Nes)‐GFP transgenic mice with C57BL/6 genetic background was provided by Dr Masahiro Yamaguchi.^28^, ^29^ Nestin‐cre mice (Stock No. 003771) and Rosa26‐td‐Tomato (R26RtdT) reporter mice (Stock No. 007905) were obtained from the Jackson Laboratory. The mice were maintained in a specific‐pathogen‐free facility, and the animal protocol was reviewed and approved by the Sun Yat‐sen University Institutional Animal Care and Use Committee.

### 2.2 Isolation and culture of specific mouse organ‐derived Nestin+ MSCs

The PPs of 3‐week‐old Nestin‐GFP mice were harvested and incubated with 10 ml of DMEM: F12 (1:1) (Gibco) digestive solution including Type IV collagenase (1 mg/ml; Gibco) and 100 IU/ml penicillin/streptomycin (Invitrogen). The tissues were homogenized and incubated at 37°C for 20 min, with shaking performed every 10 min. The resulting cell suspensions were passed through a 40‐μm cell strainer and sorted using flow cytometry (Influx, BD). The cells were cultured in DMEM: F12 (1:1) (Gibco) containing 20 ng/ml bFGF (PeproTech), 20 ng/ml EGF (PeproTech), 10 ng/ml PDGF‐BB (PeproTech), 10 ng/ml OSM (PeproTech), 1% N‐2 (Invitrogen), 2% B‐27 (Invitrogen), 0.1 mM β‐mercaptoethanol (Invitrogen), 1% nonessential amino acids (Invitrogen), 2% fetal bovine serum (FBS, Invitrogen), 100 IU/ml penicillin/streptomycin (Invitrogen) and moxifloxacin (TargetMol). Similarly, mouse femurs and tibiae were removed from peer Nestin‐GFP mice and placed on ice in 10 ml of DMEM: F12 (1:1) (Gibco). Each bone marrow cavity was flushed with medium, and individual cells were obtained by filtration through a 40‐μm cell strainer before sorting. After the red blood cells were removed by ammonium chloride‐induced lysis, the remaining cells were washed with Hanks' balanced salt solution (HBSS), resuspended in DMEM: F12 (1:1), and cultured in growth medium as described above. After 7 days or as appropriate, nonadherent cells were discarded, and the medium was replaced with fresh medium without FBS; thereafter, fresh medium was replaced every 4 days. All bone marrow‐ and PP‐derived Nestin+ cells were cultured in low‐adherence dishes (Corning) at 37°C in a 5 water‐jacketed incubator with to allow clonal sphere formation.^30^ These cells were passaged every 4–6 days, and cells from similar passages were used in all assays.

### 2.3 Cell culture

The PDT was determined using the formula Δ*t*(*h*) × log2/(log*N*_*t*_/*N*_*o*_), where Δ*t* is the culture time (hours), *N*_*t*_ is the number of harvested cells and *N*_*o*_ is the initial number of cells. A CCK‐8 kit assay (Beyotime, China) was performed according to the manufacturer's instructions. MSCs from different sources were incubated in 96‐well plates at a density of 3 × 10^3^ cells/well and evaluated at 24, 48, 72, 96 and 120 h.

### 2.4 Flow cytometric analysis

Flow cytometric analyses were performed with Influx or Gallios (Beckman Coulter) flow cytometers. The data were analysed with FlowJo v10 software (TreeStar) or CytExpert2.3 software (Beckman Coulter). The following anti‐mouse antibodies were used: CD44‐PE (IM7), CD73‐APC (AD2), CD90‐PE (30‐H12), CD106‐Alexa Fluor 647 (429), CD45‐PE‐Cyanine7 (30‐F11), CD11b‐PE‐Cyanine7 (M1/70), PDGFRα‐APC (APA5), IL‐17A‐PE‐Cyanine7 (eBio17B7), TER‐119‐PE‐Cyanine7 (TER‐119), and IL‐22‐PE (1H8PWSR) from eBioscience; CD3‐V450 (500A2), CD8‐BV510 (53–6.7), CD31‐APC (MEC 13.3), CD34‐BV421 (RAM34), CD105‐BV421 (MJ7/18) Sca‐1‐V450 (D7), TNF‐α‐PE (MP6‐XT22) and IFN‐γ‐APC (XMG1.2) from BD; and CD4‐APCCY7 (GK1.5) from BioLegend.

### 2.5 Measures of cell differentiation ability in vitro

To induce osteogenic, adipogenic, and chondrogenic differentiation, Nestin+ cells were cultured in the relevant differentiation medium for 2 ~ 3 weeks and analysed by staining with alizarin red, oil red O, and toluidine blue, respectively, as previously described.^31^ The expression levels of lineage‐specific genes (SPARC and Runx2 for osteogenesis, FabP4 and PPAR‐γ for adipogenesis, and Collagen II and Collagen X for chondrogenesis) were analysed by qPCR. The following primers were used:

SPARC forward, 5′‐TTGGCGAGTTTGAGAAGGTATG‐3′;

SPARC reverse, 5′‐GGGAATTCAGTCAGCTCGGA‐3′;

Runx2 forward, 5′‐CGTGGCCTTCAAGGTTGTA‐3′;

Runx2 reverse, 5′‐GCCCACAAATCTCAGATCGT‐3′;

FabP4 forward, 5′‐AATCACCGCAGACGACA‐3′;

FabP4 reverse, 5′‐GTGGAAGTCACGCCTTTC‐3′;

PPAR‐γ forward, 5′‐CTGACCCAATGGTTGCT‐3′;

PPAR‐γ reverse, 5′‐CAGACTCGGCACTCAATG‐3′;

Col2a1 forward, 5′‐AGTACCTTGAGACAGCACGAC‐3′;

Col2a1 reverse, 5′‐AGTCTCCGCTCTTCCACTCG‐3′;

Col10a1 forward, 5′‐CAGCAGCATTACGACCCAAG‐3′; and.

Col10a1 reverse, 5′‐CCTGAGAAGGACGAGTGGAC‐3′.

### 2.6 Reverse transcription and real‐time qPCR

Total RNA was extracted from cultured cells using a RNeasy Mini kit (250) (QIAGEN), and equal amounts of RNA were reverse transcribed using Novo Script Plus All‐in‐One 1st Strand cDNA Synthesis SuperMix (Novoprotein). The generated cDNA was subjected to real‐time PCR with SYBR Green reagent (Roche) using the following mouse primers:

GAPDH forward, 5′‐ACCACAGTCCATGCCATCAC‐3′; GAPDH reverse, 5′‐TCCACCACCCTGTTGCTGTA‐3′; IL‐22 forward, 5′‐TCCGAGGAGTCA GTGCTAAA‐3′; and IL‐22 reverse, 5′‐AGA ACGTCTTCCAGGGTGAA‐3′.

The other primers are mentioned above. Relative mRNA abundance was calculated using the Δ*Ct* or ΔΔ*Ct* method, and the gene expression level was normalized to that of GAPDH.

### 2.7 Animal model mice

We referred to a previously published protocol to induce an inflammatory bowel disease model with 7‐week‐old male C57BL/6 mice.^32^ In brief, on day one, mice were covered with 150 μl of a premixed solution containing picrylsulfonic acid (also known as trinitrobenzenesulfonic acid, TNBS, Sigma). On day seven, food but not water was withdrawn from the C57BL/6 mice (5 mice/group) for 24 h, after which the mice were anaesthetised with pentobarbital, and a 3.5‐French catheter was inserted into the colon 4 cm proximal to the anus. Subsequently, TNBS (2.5 mg in 100 μl of 50% ethanol) was delivered to the lumen using a 1‐ml syringe. The mice were maintained in an inverted pendulum for 1 min to ensure the proper distribution of the TNBS within the colon. The control mice received 100 μl of a 0.9% NaCl solution. Eight hours after treatment, 5 × 10^5^ bone marrow‐ and PP‐derived Nestin+ MSCs were delivered to mice by intravenous injection. The colons were collected and analysed 24 h after this cell infusion. All animal studies were carried out in accordance with the guidelines of the Sun Yat‐sen University Institutional Animal Care and Use Committee. For the assessment of colitis severity, animals were monitored daily for survival, body weight, and diarrhoea. The colons were measured for length without the cecum and evaluated for macro‐ or microscopic damage. Disease activity and histological scores were evaluated as previously described.^33^ MPO activity was assessed with an MPO kit (Cusabio) according to the manufacturer's instructions. IFN‐γ, TNF‐α and IL‐17 were measured by ELISA (R&D Systems; Cusabio) according to the corresponding manufacturer's instructions.

### 2.8 Cytokine assays

For in vitro cytokine assays, mouse lymphocytes were stimulated with phorbol 12‐myristate 13‐acetate (PMA; 50 ng/mL) and ionomycin (1 μg/ml) for 5 h; during this period, brefeldin A (BFA; 10 μg/ml) was used to inhibit the secretion of cytokines (all from Sigma Aldrich). IFN‐γ, TNF‐α and IL‐17 were analysed by flow cytometry. Mouse splenocytes were cultured with MSCs (30:1) or alone for 2 days before examination.

Colons from each experimental group were collected and cut into small pieces and then resuspended in 10 ml of DMEM:F12 (1:1) (Gibco) with digestive solution including Type IV collagenase (1 mg/ml; Gibco) and 100 IU/ml penicillin/streptomycin (Invitrogen) for 20 min at 37°C with shaking performed every 10 min. The resulting cell suspensions were passed through a 40 μm cell strainer and washed with PBS several times. The T cell phenotype and inflammatory cytokines were subsequently identified through flow cytometry.

### 2.9 Proliferation assays

Mouse MSCs were plated in a 48‐well plate (Corning) and cultured for 24 h before use in a lymphocyte proliferation assay. Pure splenic CD3+ T cells were sorted by flow cytometry (Influx), and 5,6‐carboxyfluorescein diacetate succinimidyl ester (CFSE; Invitrogen) staining (5 μmoL/L) was performed to assess CD3+ T cell proliferation. The cells were then suspended in Roswell Park Memorial Institute (RPMI) 1640 at 2.5 × 10^6^ cells/ml and seeded 48‐well plates (0.5 ml/well) in the presence or absence of MSCs. To induce T cell proliferation, anti‐mouse CD3 and CD28 antibodies (BD Pharmingen; final concentration, 1 μg/ml) were added to the wells. After 3 days of coculture, the CD3+ T cells were collected and analysed by flow cytometry.

### 2.10 Immunofluorescence staining

For the immunofluorescence assay, cultured cells were fixed in 4% paraformaldehyde (PFA) for 20 min, blocked for 40 min with normal goat serum, incubated with primary antibodies against ER‐TR7, PDPN, Nestin (eBioscience), IL‐22(R&D) overnight at 4°C, and then incubated with goat anti‐mouse immunoglobulin G (H + L) highly cross‐adsorbed secondary antibody, Alexa Fluor 488 and donkey anti goat AF594 secondary antibody (diluted 1:400) in the dark at room temperature for 1 h. Subsequently, all slides were stained with 300 nM DAPI (D1306, Invitrogen; Thermo Fisher Scientific, Inc.) for 5 min at room temperature. For each sample, three random fields were observed by laser scanning confocal microscopy (LSCM) at ×200 magnification. The relative fluorescence intensity was estimated with ZEN 2.6 (blue version) software.

### 2.11 RNA sequencing

To examine the biological properties of the MSCs^BM^ and MSCs^PP^, we performed RNA sequencing. RNA was prepared from cultured FACS‐purified MSCs (passage 4), sequencing libraries were constructed and sequenced on a NovaSeq system (Illumina), and the sequenced fragments were mapped to the mouse reference genome and assembled using the CLC Main Workbench package (Qiagen). The median expression level of each reconstructed mRNA was estimated by calculating the fragments per kilobase of exon per million aligned reads (FPKM). The secretory protein genes were selected basing on the annotations of Uniprot database (https://www.uniprot.org/). The transcripts of genes were quantificated using TPM (Transcripts Per Kilobase Million), and the genes with expression fold changes more than 2 were defined as differentially expressed genes.

### 2.12 IL‐22 measurement

The supernatant of MSCs^BM^ and MSCs^PP^ were stored at −80 °C until use for cytokine analysis. Murine IL‐22 (BMS6022, eBioscience) was measured using an ELISA kit according to the manufacturer's instructions.

### 2.13 RNA interference

For IL‐22 knockdown, MSCs^PP^ were transfected with 100 nmoL/L siRNAs specifically targeting mouse IL‐22 or scrambled siRNA (negative control) using a commercial kit (RiboBio, Shanghai, China) and incubated for 48 h before use in experiments.

### 2.14 Western blot analysis

Cell lysates were prepared and centrifuged at 4°C. The proteins were separated by SDS–PAGE and transferred to a polyvinylidene fluoride (PVDF) membrane. The membrane was blocked with 5% nonfat milk, incubated with rabbit anti‐IL‐22 antibody (Bioss), and then incubated at room temperature with secondary antibodies. Antigen–antibody complexes were detected by enhanced chemiluminescence (Bio‐Rad).

### 2.15 Transduction of Lentivirus in vitro

MSCs were transduced with IL‐22 gene carrying‐EGFP lentivirus (rLV‐EF1a‐mIL22‐P2A‐EGFP‐WPRE) or control EGFP lentivirus (rLV‐EF1a‐ ‐EGFP‐WPRE) (Brain Case) in the presence of polybrene (8 μg/mL) for 24 h before changing to fresh media. Fluorescent cells transduced with Lentivirus were visualized after 48 h under an inverted microscope (Leica DMi8).

### 2.16 Statistical analysis

All values in the figures and text are expressed as the means ± SD. Statistical comparisons were made with Prism software (v 7.04, GraphPad) using two‐tailed Student's *t* tests (between two groups) or one‐way ANOVAs (for multigroup comparisons) as appropriate. *p* ≤ 0.05 (*) was considered statistically significant.

## 3 RESULTS

### 3.1 Isolation and characterization of Nestin+ cells in the PPs of transgenic mice

Secondary lymphoid organs with PPs consist of heterogeneous stromal cells resembling fibroblastic reticular cells (FRCs).^34^, ^35^ We first observed the relationship between Nestin and PP stromal cells with specific markers, including ER‐TR7, PDPN (Figure S1). Immunofluorescence staining indicated that Nestin positive cells exist in PPs and co‐localize with the specific marker of FRCs (Figure S1), suggesting that Nestin+ cells in PPs may be the stromal cell population of lymphoid tissues.

**FIGURE 1:**
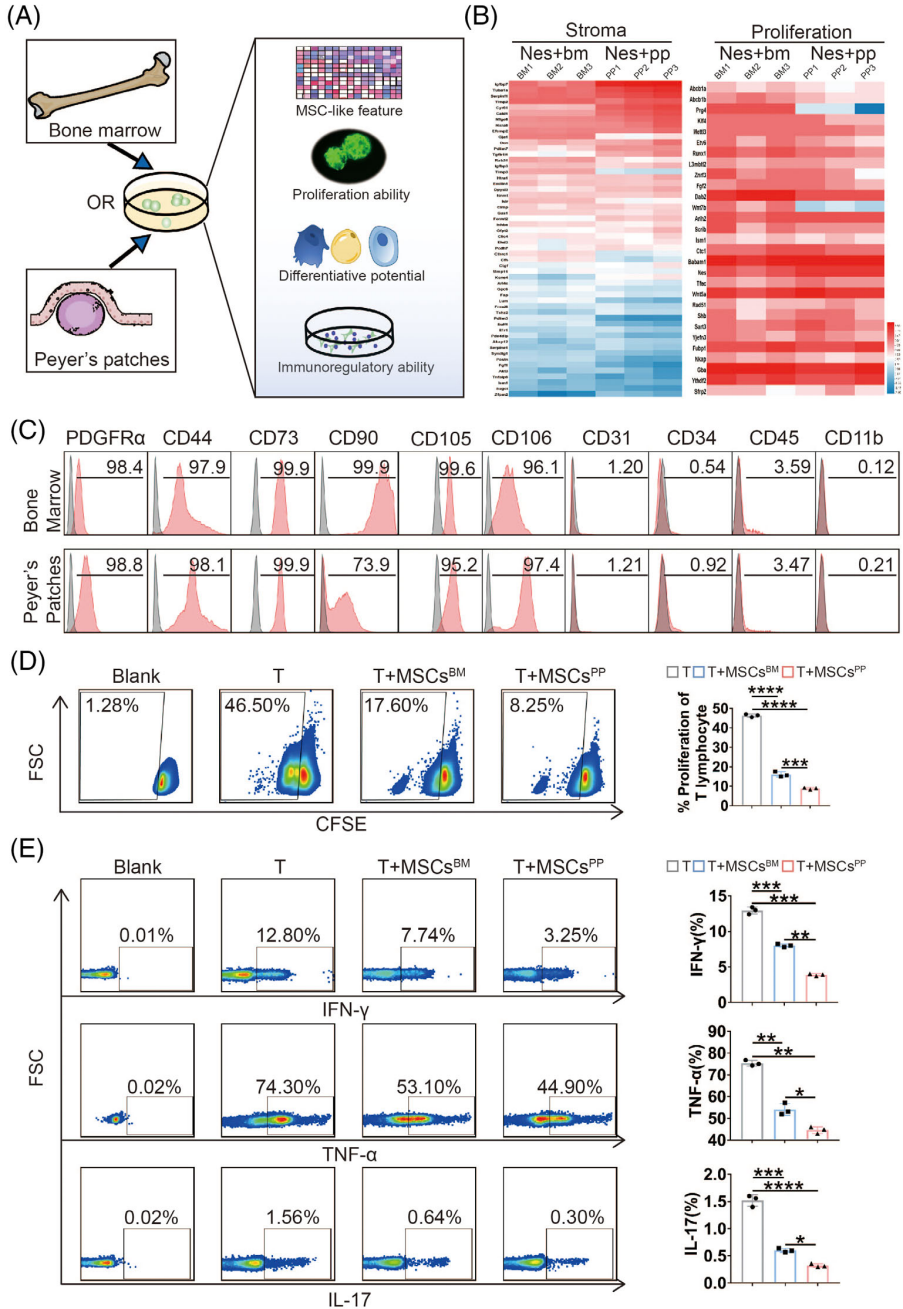
Isolation and characterization of Nestin+ cells derived from bone marrow or Peyer's patches in transgenic mice. (A) A graphical scheme of Nestin+ cell isolation. (B) Heat map showing the expression patterns of representative genes with relevant functions. (C) Expression of MSC‐related markers on bone marrow‐ and Peyer's patch‐derived Nestin+ cells. The proliferation (D) and secretion of IFN‐γ, TNF‐α, and IL‐17 (E) from splenic CD3+ T cells were analysed after T cells were cocultured with or without MSCs^BM/PP^. Data were presented as the means ± SD (*n* = 3). *p < 0.05, **p < 0.01, ***p < 0.001, ****p < 0.0001. TNF, tumour necrosis factor; IFN, interferon; IL, interleukin; MSCs^BM^, bone marrow‐derived Nes + MSCs; MSCs^PP^, Peyer's patch‐derived Nes + MSCs.

Furthermore, to verify whether PP Nestin+ stromal cells possess MSC‐like properties consistent with their bone marrow counterparts, we isolated Nestin+ cells from bone marrow and intestinal PPs of Nestin‐GFP transgenic mice (Figure 1A). RNA sequencing (RNA‐seq) analysis of the isolated Nestin+ cells revealed that Nestin+ cells derived from the tissues were similar in stromal feature and proliferation ability (Figure 1B). Next, we detected the expression of Nestin on both cells and further observed no significant difference in proliferation between them (Figure S2A‐C). Additionally, the PP‐derived Nestin+ cells expressed PDGFRα, CD44, CD73, CD90, CD105 and CD106 but not CD31, CD34, CD45 and CD11b, findings consistent with the bone marrow‐derived Nestin+ cells (Figure 1C). Moreover, similar to their bone marrow counterparts, PP‐derived Nestin+ cells could differentiate into osteocytes, adipocytes, and chondrocytes (Figure S2D). Hereto, given the similarity of MSC‐like features between bone marrow‐derived and PP‐derived Nestin+ cells, we designated these cells MSCs^BM^ and MSCs^PP^, respectively.

Subsequently, to investigate the immunomodulatory capacity between MSCs^BM^ and MSCs^PP^, we measured the effects of MSCs^BM^ and MSCs^PP^ on the proliferation and proinflammatory cytokine production of CD3+ T cells. As shown in Figure 1D, E, MSCs^PP^ showed superior immunoregulatory ability than MSCs^BM^ in inhibiting the proliferation of splenic T cells (46.75% ± 0.35% vs. 16.30% ± 1.84% vs. 8.77% ± 0.73%) and suppressing T cell secretion of proinflammatory cytokines, including interferon‐γ (IFN‐γ, 12.87% ± 0.49% vs. 7.92% ± 0.27% vs. 3.67% ± 0.40%), tumour necrosis factor α (TNF‐α, 75.37% ±1.30% vs. 54.11% ± 2.75% vs. 44.63% ± 1.52%) and IL‐17(1.52% ± 0.11% vs. 0.60% ± 0.04% vs. 0.32% ± 0.03%). CD4^+^ and CD8^+^ T cells analyses also were consistent with the results above (Figure S3A). Collectively, we isolated and identified Nestin+ MSCs in PPs and found that MSCs^PP^ exhibit more powerful immunoregulatory ability than MSCs^BM^.

### 3.2 MSCs^PP^ exhibit enhanced therapeutic potential compared with MSCs^BM^ in murine inflammatory bowel disease

**FIGURE 2:**
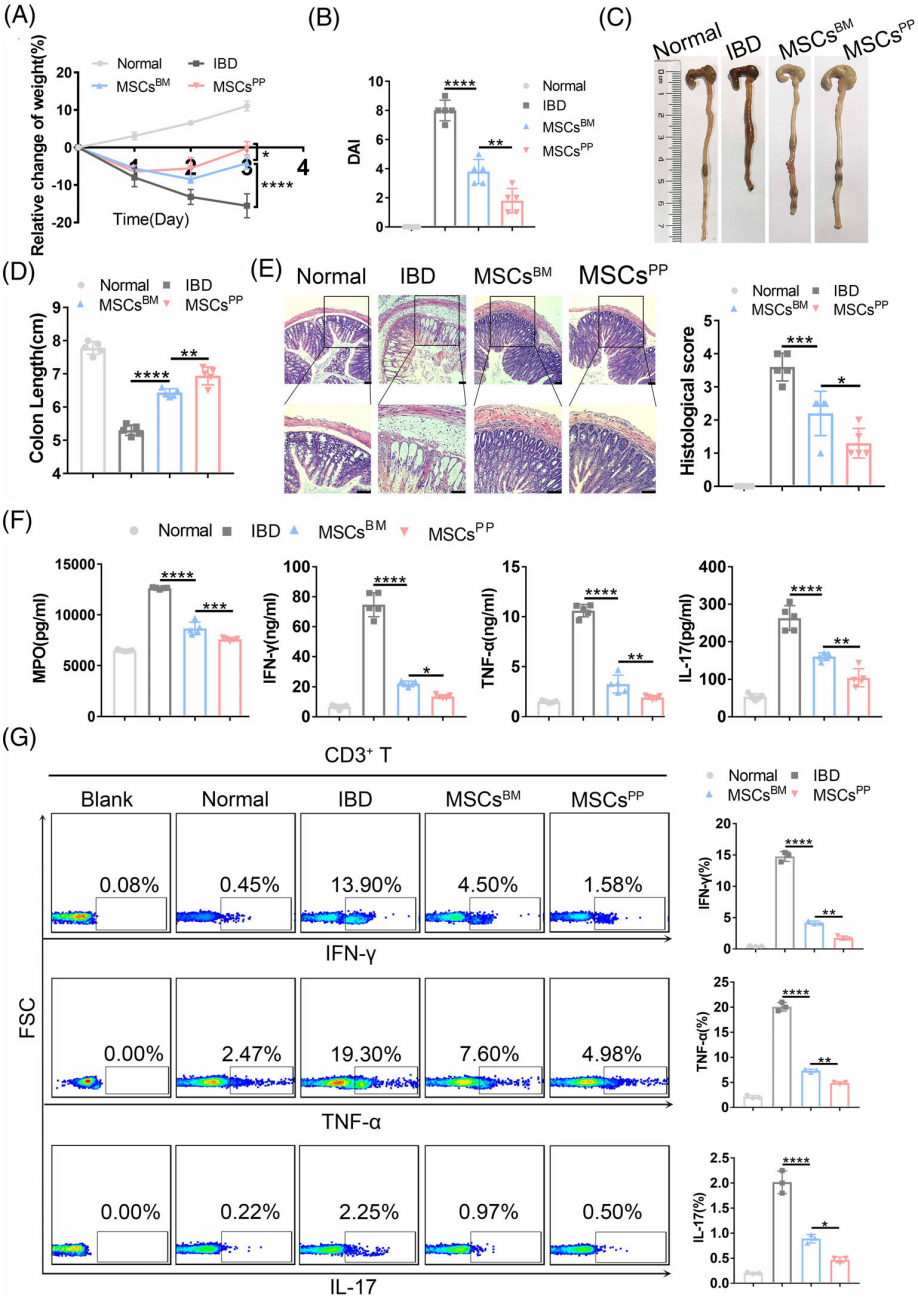
MSCs^PP^ exhibit enhanced therapeutic potential compared with MSCs^BM^ in inflammatory bowel disease. Colitis severity assessments of Normal, IBD, MSCs^BM^ and MSCs^PP^ groups are shown, including measurement of (A) body weight, (B) disease activity index, (C) evaluation of gross morphology images, (D) colon length measurements, (E) H&E staining and corresponding histological scoring statistical histogram, (F) MPO, IFN‐γ, TNF‐α and IL‐17 expression levels as measured with corresponding murine ELISA kits, and (G) inflammatory cytokine level secreted by CD3+ T cells as assessed with flow cytometry. Scale bar, 100 μm. Data were presented as the means ± SD (*n* = 3). *p < 0.05, **p < 0.01, ***p < 0.001, ****p < 0.0001 (*n* ≥ 3 mice in each group). Normal, control group treated with 0.9% NaCl; IBD, murine model group treated with TNBS; MSCs^BM^, model group treated with MSCs^BM^; MSCs^PP^, model group treated with MSCs^PP^.

Considering the unique location of PPs, we verified the therapeutic potential of MSCs^BM^ and MSCs^PP^ in murine IBD. As shown in Figure 2A–G, MSCs^PP^ that were injected intravenously into IBD model mice alleviated the severity of colonic inflammation to a much greater extent than injected MSCs^BM^. Specifically, body weight loss was reversed more quickly in the MSCs^PP^‐treated group than MSCs^BM^‐treated and IBD control group (−0.22% ± 1.80% vs. −4.20% ± 1.58% vs. −15.55% ± 3.21%, *p* < 0.05, Figure 2A). Moreover, MSCs^PP^ treatment led to a notable improvement in disease activity indexes (DAIs) compared to those of the MSCs^BM^ and IBD groups (1.8 ± 0.84 vs. 3.80 ± 0.84 vs. 8.00 ± 0.71, *p* < 0.01, Figure 2B). Gross images revealed that reduced colon length was remarkably reversed by infusion of MSCs^PP^ compared to MSCs^BM^ and 0.9% NaCl infusion (6.94 ± 0.27 vs. 6.43 ± 0.12 vs. 5.30 ± 0.16, *p* < 0.01, Figure 2C, D). MSCs^PP^ also exhibited better effects than MSCs^BM^ in term of improving the histological scores (1.30 ± 0.45 vs. 2.20 ± 0.67, *p* < 0.05, Figure 2E) and reducing the MPO activity (7571.32 ± 156.85 vs. 8661.37 ± 619.28 pg/ml, *p* < 0.001) and inflammatory cytokine levels (Figure 2F), including IFN‐γ (13.49 ± 1.22 vs. 22.02 ± 1.74 ng/ml, *p* < 0.05), TNF‐α (1.93 ± 0.18 vs. 3.27 ± 0.89 ng/ml, *p* < 0.01) and IL‐17 (103.95 ± 23.75 vs. 160.78 ± 10.29 pg/ml, *p* < 0.01), in local IBD lesions. Furthermore, MSCs^PP^ dramatically reduced the secretion of inflammatory cytokines by CD3+, CD4+ and CD8+ T cells more than MSCs^BM^ did (Figures 2G and Figure S4A). In addition, we counted the number of localized MSCs^BM^ and MSCs^PP^ but found no significant differences (Figure S4B). Collectively, these results suggest that MSCs^PP^ show better therapeutic potential than MSCs^BM^ for ameliorating murine IBD.

### 3.3 Identification of differentially expressed genes in MSCs^BM^ and MSCs^PP^

**FIGURE 3:**
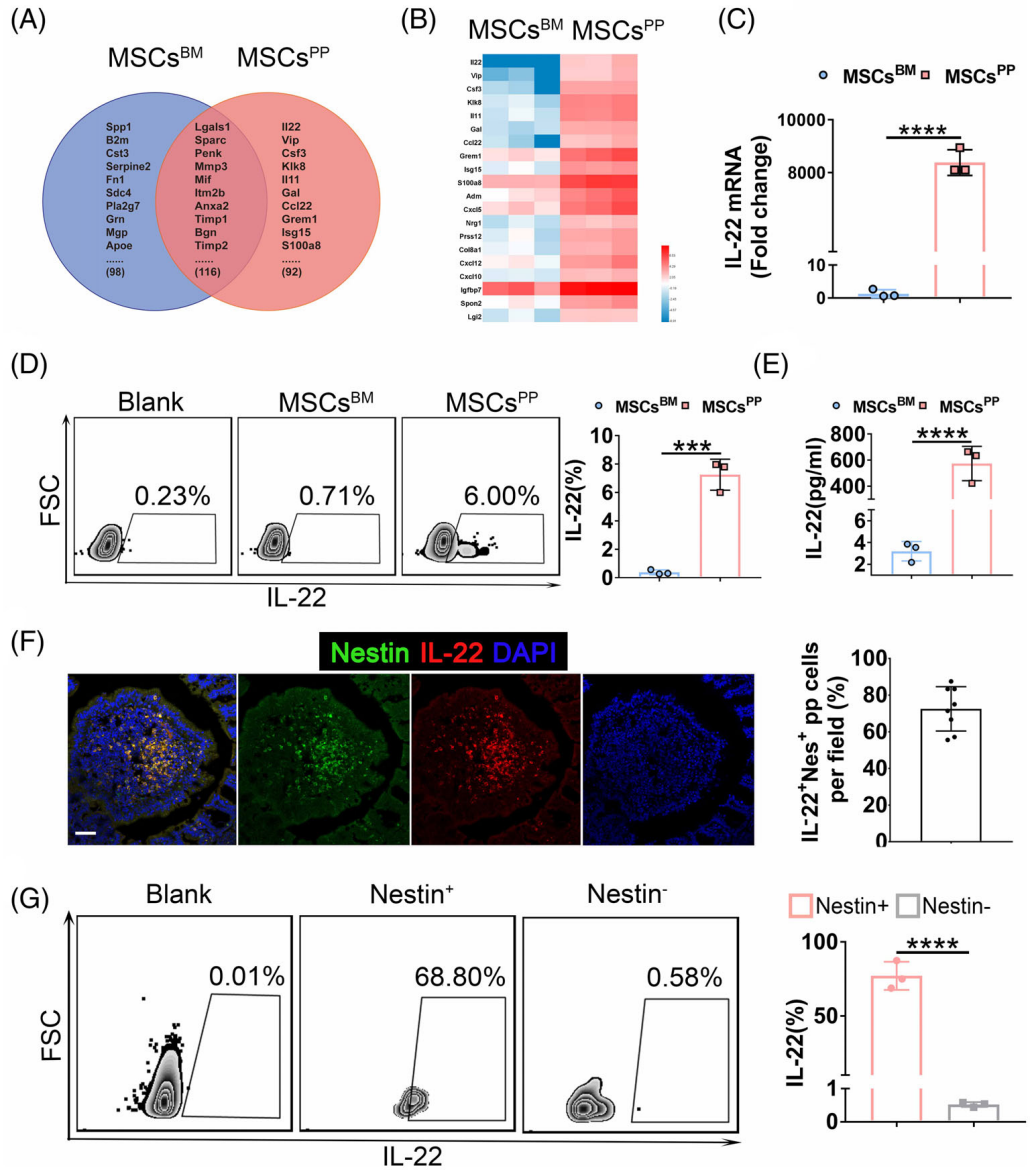
Expression profile of MSCs^BM^ and MSCs^PP^. (A) Venn diagram of the secretory proteins expressed in MSCs^BM^ and MSCs^PP^. (B) The 20 most significant different secretory proteins in MSCs^PP^ compared with the expression of their counterparts in MSCs^BM^. (C–E) The expression level of IL‐22 in MSCs^BM^ and MSCs^PP^ was confirmed by qPCR, flow cytometry and ELISA kit. (F) Immunofluorescence analysis of IL‐22 in Peyer's patches of Nestin‐GFP transgenic mice. IL‐22 + Nes + pp cells in each microscopic field were quantified and plotted in a histogram. Scale bars, 50 μm. (G) Flow cytometry analyses of CD45‐Ter119‐CD31‐IL‐22+ cells in Peyer's patches of Nestin‐GFP transgenic mice. Data were presented as the means ± SD (*n* = 3). ***p < 0.001, ****p < 0.0001. MSCs^BM^, bone marrow‐derived Nes + MSCs; MSCs^PP^, Peyer's patch‐derived Nes + MSCs.

To further explore the underlying mechanisms by which murine MSCs^PP^ exhibit enhanced therapeutic capability in murine IBD, an unbiased transcriptome analysis strategy was employed. Heat maps revealed 306 expressed genes of secretory proteins in MSCs^BM^ and MSCs^PP^ (Figure S5A). In total, 92 more highly expressed secretory proteins in MSCs^PP^ and 98 more highly expressed secretory proteins in MSCs^BM^ showed unique MSC‐specific expression patterns (Figure 3A). Complementarily, a Gene Ontology (GO) term analysis suggested that 92 highly expressed secretory proteins in MSCs^PP^ or 98 highly expressed secretory proteins in MSCs^BM^ are involved in the immune response, signal transduction, cell communication, protein metabolism, etc. The 20 most significant different secretory proteins in MSCs^PP^, compared with their counterparts expressed in MSCs^BM^, provided further evidence to explain the distinct immunoregulatory ability of MSCs^PP^ (Figure 3B). Moreover, we found that the expression of secretory proteins linked to intestinal homeostasis, including IL‐22,^36^, ^37^ Vip,^38^ Csf,^39^ Grem1,^40^ S100a8^41^ and Igfbp7,^31^ was significantly higher in the MSCs^PP^. In particular, IL‐22 was negligibly expressed in MSCs^BM^. Hence, IL‐22 expression was measured at the mRNA and protein levels (Figure 3C–E). These results were consistent with the RNA‐seq data. Furthermore, performing immunofluorescence staining and flow cytometry, we confirmed that nearly 80% of the IL‐22‐positive cells in PPs were in the Nestin+ cells of the Nestin‐GFP transgenic mice (Figure 3F, G). Taken together, these results showed that the transcriptomes in MSCs^BM^ and MSCs^PP^ differed, which hint that the most differentially expressed gene, IL‐22, likely explains, to some degree, the difference in the therapeutic potential of MSCs^BM^ and MSCs^PP^ with respect to murine IBD.

### 3.4 MSCs^PP^ alleviate murine IBD partially through IL‐22

**FIGURE 4:**
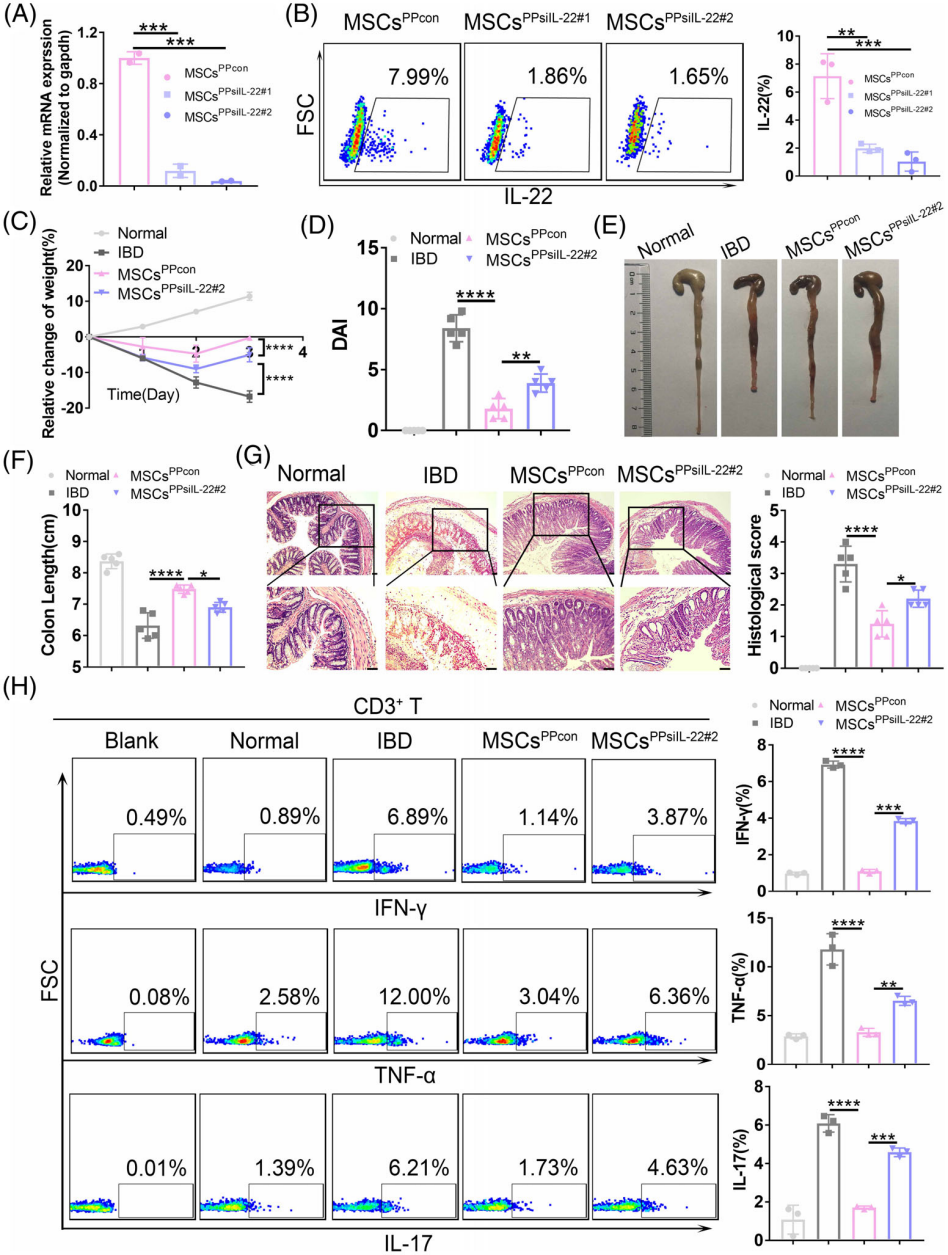
Knockdown of IL‐22 in MSCs^PP^ by RNA interference. (A) The efficiency of siRNA‐mediated downregulation of IL‐22 was assessed at the RNA level and normalized with respect to the expression of gapdh. (B) Flow cytometry analysis of IL‐22 knockdown at the protein level. Colitis assessments including (C) body weight measurement, (D) disease activity index, (E) evaluation of gross morphology images, (F) colon length measurements, (G) H&E staining and corresponding histological scoring statistical histogram, and (H) inflammatory cytokine expression in CD3+ T cells detected by flow cytometry. Scale bar, 100 μm. Data were presented as the means ± SD (*n* = 3). *p < 0.05, **p < 0.01, ***p < 0.001, ****p < 0.0001 (*n* ≥ 3 mice in each group). Normal, control group treated with 0.9% NaCl; IBD, murine model group treated with TBNS; MSCs^PPcon^, model group treated with control MSCs^PP^; MSCs^PPsiIL‐22#2^, model group treated with IL‐22‐knockdown MSCs^PP^.

Considering that it can stimulate mucus production and promote goblet cell restitution under inflammatory conditions, IL‐22 is widely recognized as an epithelium protection factor that contributes to intestinal homeostasis.^36^, ^37^ To determine whether IL‐22 participates in the immunomodulatory activity of MSCs^PP^, we knocked down IL‐22 in MSCs^PP^ using two different target short interfering RNA (siRNA) sequences (MSCs^PPsiIL‐22#1^ and MSCs^PPsiIL‐22#2^). Compared with control MSCs^PP^ (MSCs^PPcon^) transduced with fixed but untargeted sequences, both MSCs^PPsiIL‐22^ expressed lower levels of IL‐22, as evidenced by qRT‐PCR, flow cytometry and western blot (Figures 4A, B and S6A). We chose MSCs^PPsiIL‐22#2^ for the following experiments due to its lower expression of IL‐22, and confirmed that MSCs^PPsiIL‐22#2^ showed less effective therapeutic effects in murine IBD models than MSCs^PPcon^ on the basis of colitis evaluation parameters, including body weight, DAI, colon length, histological scores and inflammatory cytokines. Specifically, body weight loss was reversed more quickly in MSCs^PPcon^‐treated mice than in MSCs^PPsiIL‐22#2^‐treated mice (−0.23% ± 0.79% vs. −5.01% ± 1.96%, *p* < 0.001, Figure 4C). In addition, MSCs^PPcon^ treatment led to a conspicuous improvement in DAIs compared to MSCs^PPsiIL‐22#2^ treatment (1.8 ± 0.83 vs. 3.90 ± 0.74, *p* < 0.01, Figure 4D). Gross images showed that the reduced colon length in colitis was strikingly attenuated by MSCs^PPcon^ infusion compared with MSCs^PPsiIL‐22#2^ infusion (7.48 ± 0.13 vs. 6.90 ± 0.16, *p* < 0.05, Figure 4 E, F). Through the histopathologic analysis, we observed that epithelial destruction and scattered infiltration were attenuated to a greater extent in the MSCs^PPcon^ group than in the MSCs^PPsiIL‐22#2^ treatment group (1.40 ± 0.42 vs. 2.20 ± 0.27, *p* < 0.05, Figure 4G). In addition, colon IFN‐γ, TNF‐α, and IL‐17 expression was also distinctly reduced in the MSCs^PPcon^ group than in the MSCs^PPsiIL‐22#2^ treatment group (Figures 4H and S6B). All these results suggested that IL‐22 may play a unique role in the enhanced therapeutic potential of MSCs^PP^ compared with MSCs^BM^ in murine IBD.

### 3.5 Recombinant IL‐22 (rIL‐22) effectively relieves the severity of murine IBD

**FIGURE 5:**
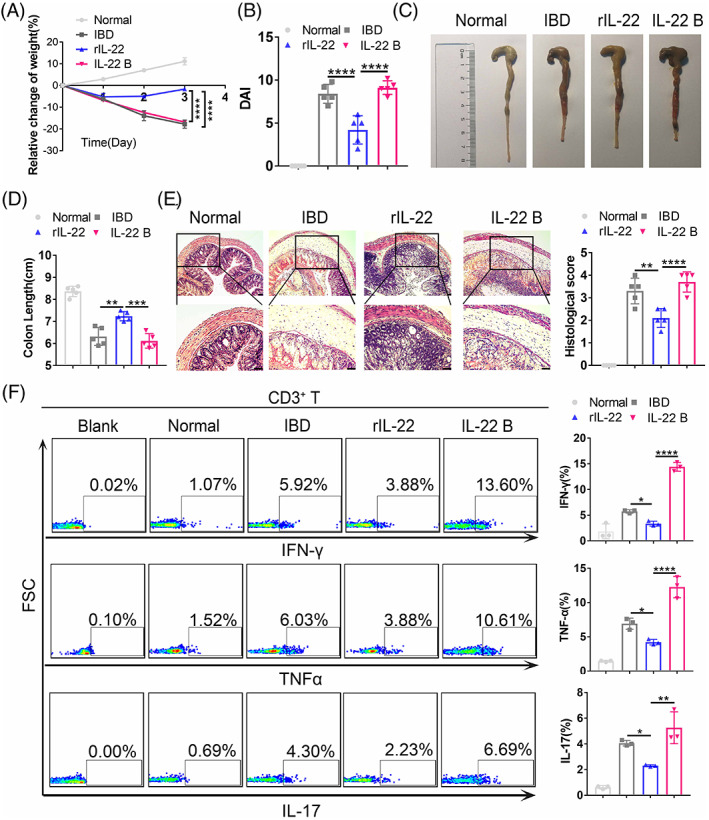
MSCs^PP^ alleviate murine IBD partially through IL‐22. Colitis assessments included measurement of (A) body weight, (B) disease activity index, (C) evaluation of gross morphology images, (D) colon length measurement, (E) H&E staining and corresponding histological scoring statistical histogram, and (F) inflammatory cytokine expression in CD3+ T cells as assessed with flow cytometry. Scale bar, 100 μm. Data were presented as the means ± SD (*n* = 3). *p < 0.05, **p < 0.01, ***p < 0.001, ****p < 0.0001 (*n* ≥ 3 mice in each group). Normal, control group treated with 0.9% NaCl; IBD, murine model group treated with TNBS; rIL‐22, model group treated with recombinant IL‐22 protein; IL‐22 B, model group treated with the IL‐22‐blocking antibody.

Additionally, to verify that IL‐22 plays an essential role in relieving the inflammatory response of colitis mice, IBD mice received intravenous injection of recombinant IL‐22 protein (rIL‐22) and anti‐IL‐22 antibody that neutralizes IL‐22 by blocking its expression (IL‐22 B). As expected, compared with the effect in the IBD model group, rIL‐22 effectively alleviated the severity of the intestinal inflammatory response and tissue damage and promoted rapid tissue repair in the IBD group. In contrast, compared with the rIL‐22 treatment group, the anti‐IL‐22 antibody that blocks and neutralizes the IL‐22 expression notably elevated IFN‐γ, TNF‐α, and IL‐17 expression and showed more severe IBD symptoms (Figure 5A–F). Specifically, it was exhibited on body weight loss (−17.70% ± 1.89% vs. −1.65% ± 0.16% vs. −16.81% ± 0.49%, *p* < 0.0001), DAIs (8.40 ± 1.10 vs. 4.20 ± 1.64 vs. 9.12 ± 0.79, *p* < 0.0001), colon length (6.32 ± 0.41 vs. 7.24 ± 0.21 vs. 6.12 ± 0.33, *p* < 0.0001) and histopathologic score (3.30 ± 0.57 vs. 2.10 ± 0.42 vs. 3.70 ± 0.45, *p* < 0.0001). In summary, our results indicated that IL‐22 may be a promising molecule to target in the treatment of murine IBD.

### 3.6 IL‐22 overexpressing MSCs^BM^ significantly enhance therapeutic potency

**FIGURE 6:**
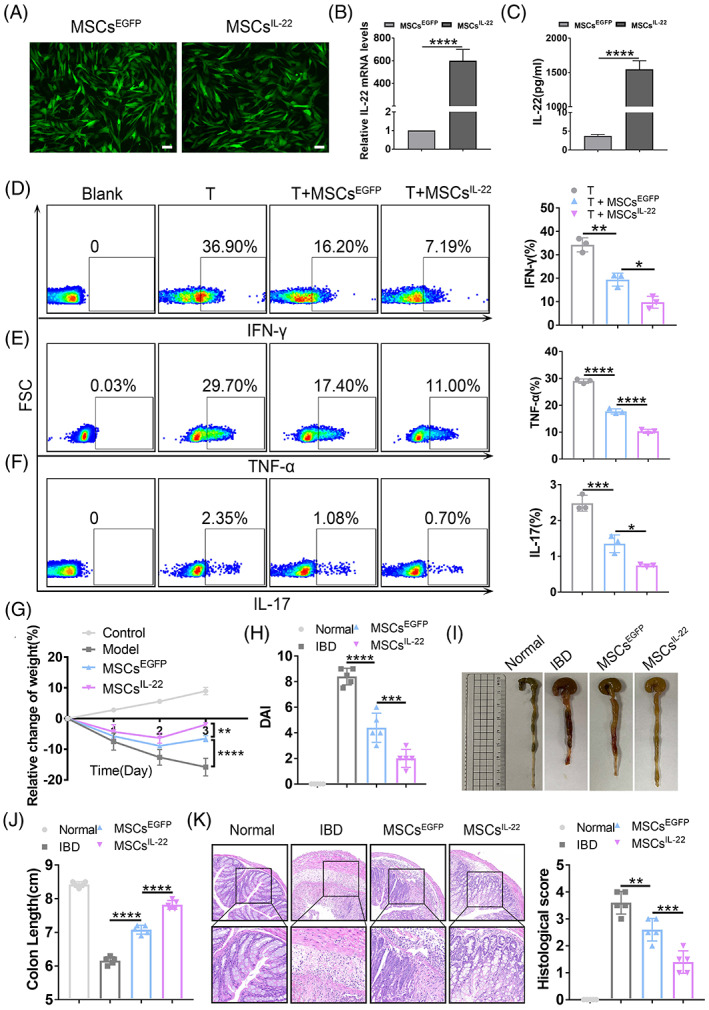
IL‐22 overexpressing MSCs^BM^ enhance the therapeutic potency. (A) The microscopical observation of MSCs^EGFP^ and MSCs^IL‐22^ which transduced with control EGFP lentivirus and IL‐22 gene carrying‐GFP lentivirus, respectively, after 48 h. (B) The mRNA level of IL‐22 on MSCs^IL‐22^ and MSCs^EGFP^. (C) The protein level of IL‐22 on MSCs^IL‐22^ and MSCs^EGFP^ detected by ELISA. The secretion of (D) IFN‐γ, (E) TNF‐α, and (F) IL‐17 from splenic CD3+ T cells were analysed after T cells were cocultured with or without MSCs^EGFP/IL‐22^. Murine IBD severity assessments of Normal, IBD, MSCs^EGFP^ and MSCs^IL‐22^ groups are shown, including measurement of (G) body weight loss, (H) disease activity index, (I) gross morphology images, (J) colon length measurements, (K) H&E staining analysis and corresponding histological scoring statistical histogram. Scale bars, 75 μm. Data were presented as the means ± SD (*n* = 3). *p < 0.05, **p < 0.01, ***p < 0.001, ****p < 0.0001 (*n* ≥ 3 mice in each group).

Although PP‐derived MSCs exhibit superior therapeutic potency in the treatment of IBD, there are limitations in obtaining MSCs from PP tissue for clinical applications. Due to the attractive effect of IL‐22 in treating murine IBD model, we then try to develop strategies to enhance the therapeutic potency of readily obtainable MSCs, such as BM‐derived MSCs. Therefore, murine MSCs^BM^ were transduced with the control EGFP lentivirus (MSCs^EGFP^) or IL‐22 gene carrying‐EGFP lentivirus (MSCs^IL‐22^) and MSCs^IL‐22^ showed significantly higher expression of IL‐22 than those of MSCs^EGFP^ (*p* < 0.0001, Figure 6A–C). We found proinflammatory cytokines secreted by T cells were significantly inhibited in MSCs^IL‐22^ group compared with MSCs^EGFP^ group, including interferon‐γ (IFN‐γ, 9.78% ± 2.56% vs. 19.40% ± 2.78%, *p* < 0.05, Figure 6D), tumour necrosis factor α (TNF‐α, 10.30% ±0.75% vs. 17.73% ± 0.95%, *p* < 0.0001, Figure 6E) and IL‐17 (0.74% ± 0.04% vs. 1.35% ± 0.25%, *p* < 0.05, Figure 6F). Meanwhile, MSCs^IL‐22^ markedly alleviated the severity of murine IBD compared with MSCs^EGFP^. Specifically, body weight loss was reversed more quickly in the MSCs^IL‐22^‐treated group than MSCs^EGFP^‐treated group (Figure 6G). Disease activity indexes (DAI) were lower in the MSCs^IL‐22^‐treated group than MSCs^EGFP^‐treated group (2.00 ± 0.71 vs. 4.40 ± 1.14, *p* < 0.001, Figure 6H). Gross images revealed shortened colons were remarkably reversed by infusion of MSCs^IL‐22^ compared with MSCs^EGFP^ (7.82 ± 0.13 cm vs. 7.08 ± 0.13 cm, *p* < 0.0001, Figure 6I, J). Moreover, MSCs^IL‐22^ treatment led to a more notably improvement in attenuating the destruction of epitheliums and inflammatory infiltration in the pathological colon, compared with those of MSCs^EGFP^ (1.40 ± 0.42 vs. 2.60 ± 0.42, *p* < 0.001, Figure 6K). Hence, IL‐22 overexpressing MSCs^BM^ could significantly improve their therapeutic potency, which is of great significant of further promote the curative effect of MSCs in clinical practice.

## 4 DISCUSSION

The discrepant clinical results suggested that the functional heterogeneity of MSCs depends upon the tissue source. MSCs were originally isolated from bone marrow,^42^ and were later found in the umbilical cord and adipose tissue.^43^, ^44^ More importantly, MSCs originating from a specific tissue may exert additive effects on the repair of the corresponding tissue. As our research group previously reported, testis‐derived MSCs can partially reestablish testosterone production and thus enhance spermatogenesis^28^; kidney‐derived MSCs participate in renal injury repair^29^; cardiac MSCs show superior potential for myocardium repair compared to nonresident MSCs.^21^ Hence, in addition to the aforementioned MSCs, we wondered whether MSCs from secondary lymphoid organs, such as PP‐derived MSCs, can be optimal candidates for IBD treatment.

In this study, we first isolated Nestin+ cells from murine PPs, and the identified MSCs^PP^ showed some similarities in cell surface antigen profile, proliferation capacity, multilineage differentiation potential and transcriptome with MSCs^BM^. Nonetheless, we noted that phenotypic profiles between murine MSCs^PP^ and MSCs^BM^ were not exactly identical, particularly CD90. *Li*. et al identified CD90^high^ and CD90^low^ murine adipose‐derived mesenchymal stem cells (ADSCs) subsets and proposed CD90^high^ ADSCs could be converted into CD90^low^ ADSCs by stimulation with LPS.^45^ Compared with BM, MSCs in intestine PPs have more chance to contact with gut microbiota and can be exposed to LPS, which might result in the low expression of CD90. Therefore, tissue‐heterogeneity from different origins since embryonic development and special microenvironment simultaneously shaped the phenotype of individual stromal cells fundamentally. Meanwhile, we also observed that MSCs^PP^ have better chondrogenic differentiation potency than MSCs^BM^. The reason may be explained by their higher expression of chondrogenic related genes according to our bulk RNA‐seq data, such as TGFβ1, TGFβ3, BMP2 and ITGB1^46^, ^47^, ^48^, ^49^ (Figure S7A). Ingenuity Pathway Analysis also identify the similar point that both of these genes are related to cartilage growth and development (Figure S7B).

The interaction between lymphoid progenitor cells and stromal cells in PPs plays an important role in the maintenance of intestinal immune homeostasis.^22^, ^23^, ^24^, ^25^ Lymphoid tissue organizer (LTO) cells can differentiate into a variety of lymphatic stromal cell components, secreting the cytokines BAFF and APRIL required for B cell survival, contributing to the stability of high endothelial cell veins, and maintaining the mobilization and survival of T cells.^23^, ^24^, ^50^ MSCs obtained from tissues in situ are usually localized to a specific tissue microenvironment where they are thought to contribute to tissue homeostasis.^21^, ^51^ Among these cells, FRCs have a strong immunosuppressive function, which includes clearing autoreactive T cells, promoting tolerance, inhibiting T cell proliferation and promoting regulatory T cell (Treg) proliferation, to prevent the occurrence of autoimmune diseases, similar to the immune regulatory function of MSCs. Fletcher Al et al. showed that FRCs isolated from lymph nodes showed much better therapeutic efficacy than bone marrow‐derived MSCs in a mouse sepsis model.^52^ Our results showed PP‐derived MSCs most likely co‐localizing with the specific marker of FRCs, also suggested that MSCs^PP^ contributed to tissue homeostasis and may have greater immunomodulatory ability.

In view of the possible mechanisms involved in MSCs^PP^ treating murine IBD, we further applied RNA‐sequencing to reveal the heterogeneity between these two tissues‐derived MSCs and demonstrated that IL‐22 played an important role in the enhanced therapeutic effects of MSCs^PP^. The RNA‐seq data confirmed that MSCs^PP^ express higher levels of antiapoptotic and survival genes (Grem1 and VEGF), immunoregulatory genes (IGFBP7 and FGF7), and intestinal homeostasis‐related genes (IL‐22, VIP, CSF and S100a8) than MSCs^BM^. Specifically, IGFBP7 is closely related to the suppression of T‐cell proliferation, cell cycle and cytokine production, all of which plays dominant roles in IBD,^31^ while Mfge8 has been reported to be an important molecule in maintaining immune homeostasis.^53^, ^54^ Our study showed that Mfge8 is highly expressed in MSCs^PP^ and indicated that recombinant Mfge8 protein has distinct therapeutic effects on murine IBD compared with neutralized Mfge8 (data not shown). However, Mfge8 exerted less effective therapeutic effects in murine IBD treatment compared with IL‐22. IL‐22, as a member of the IL10 family of cytokines, has been implicated in multiple aspects of epithelial barrier function, including the regulation of epithelial cell growth and permeability and the production of mucus and antimicrobial proteins.^55^, ^56^, ^57^, ^58^, ^59^ Preclinical data have suggested that IL‐22 or IL‐22 receptor deficiency leads to exacerbated dextran sulphate sodium (DSS)‐induced colitis or T cell‐induced colitis.^32^ In contrast, either IL‐22 or an IL‐22‐Fc fusion protein is efficacious in attenuating DSS‐induced colitis in model mice.^60^ These results were consistent with those of previous reports, further corroborating the evidence of the enhanced therapeutic capability of MSCs^PP^ through IL‐22. Christian B. Cox et al. proposed that IL1R1‐dependent induction of IL‐22 in innate lymphoid cells (ILCs) contributes to host resistance and epithelial regeneration and that GREM1+ mesenchymal cells are required for crypt protection after *Citrobacter rodentium* infection.^40^ Our research revealed that MSCs^PP^ not only express IL‐22 but also highly express GREM1, which further shows their advantages in murine IBD treatment.

Although PP‐derived MSCs exhibited superior therapeutic potency in the treatment of murine IBD, there are limitations in obtaining MSCs from PP tissue for clinical applications. Therefore, elucidating the therapeutic mechanisms of MSCs^PP^ is helpful to identify the functional molecules, which can be used to develop strategies to enhance the therapeutic potency of readily obtainable MSCs, such as BM‐ or adipose‐derived MSCs. We overexpressed IL‐22 in MSCs^BM^, which can significantly improve the symptoms of murine experimental IBD model, ameliorate the severity of induced colonic histopathological inflammation and restore the injured gastrointestinal mucosal tissues. Here, we proposed the potential on research findings on tissue‐specific MSCs should be translated to develop strategies to improve the therapeutic potency of readily obtainable MSCs.

In summary, we prospectively identified murine PP‐derived Nestin+ cells possessing MSC‐like properties and demonstrated Nestin is a suitable marker for MSCs^PP^. Furthermore, we found MSCs^PP^ showed superior efficacy than nonresident MSCs partly through IL‐22 in murine IBD treatment. Thus, this study reveals optimal source of MSCs for clinical indications and provides new strategy modified readily obtainable MSCs in IBD therapy.

## AUTHOR CONTRIBUTIONS

Conceptualization, Andy Peng Xiang and Xiaoran Zhang; methodology, Jieying Chen, Jing Huang, Qiaojia Li, Erming Zhao, Gang Li, Jiahao Shi, Wenzhe Mao, Rui Fang; investigation, Xiaoran Zhang, Jieying Chen, Jing Huang, Jiahao Shi, Minrong Li, and Xiaoyong Chen; writing—original draft, Jieying Chen and Xiaoran Zhang; writing—review & editing, Andy Peng Xiang and Xiaoran Zhang; funding acquisition, Andy Peng Xiang, Xiaoyong Chen, and Jianping Ma; resources, Weijun Huang, Weiqiang Li, Jing Huang; and supervision, Andy Peng Xiang

## CONFLICT OF INTEREST

The authors declare no conflict of interest.

## Supporting information


**APPENDIX S1:** Supplementary Figures

**APPENDIX S2:** Supporting Information

## Data Availability

The datasets generated and/or analysed during the current study are available from the corresponding author upon reasonable request.
