# Supplementary material for: Nestin+ Peyer's patch resident MSCs enhance healing of inflammatory bowel disease through IL‐22‐mediated intestinal epithelial repair
Source: Cell Prolif. 2022 Nov 20;56(2):e13363. doi: 10.1111/cpr.13363 (PMC9890526; doi:10.1111/cpr.13363)
Supplement: Supplementary file 1 — APPENDIX S1: Supplementary Figures [file CPR-56-e13363-s001.docx]

**Supplementary Materials**

**
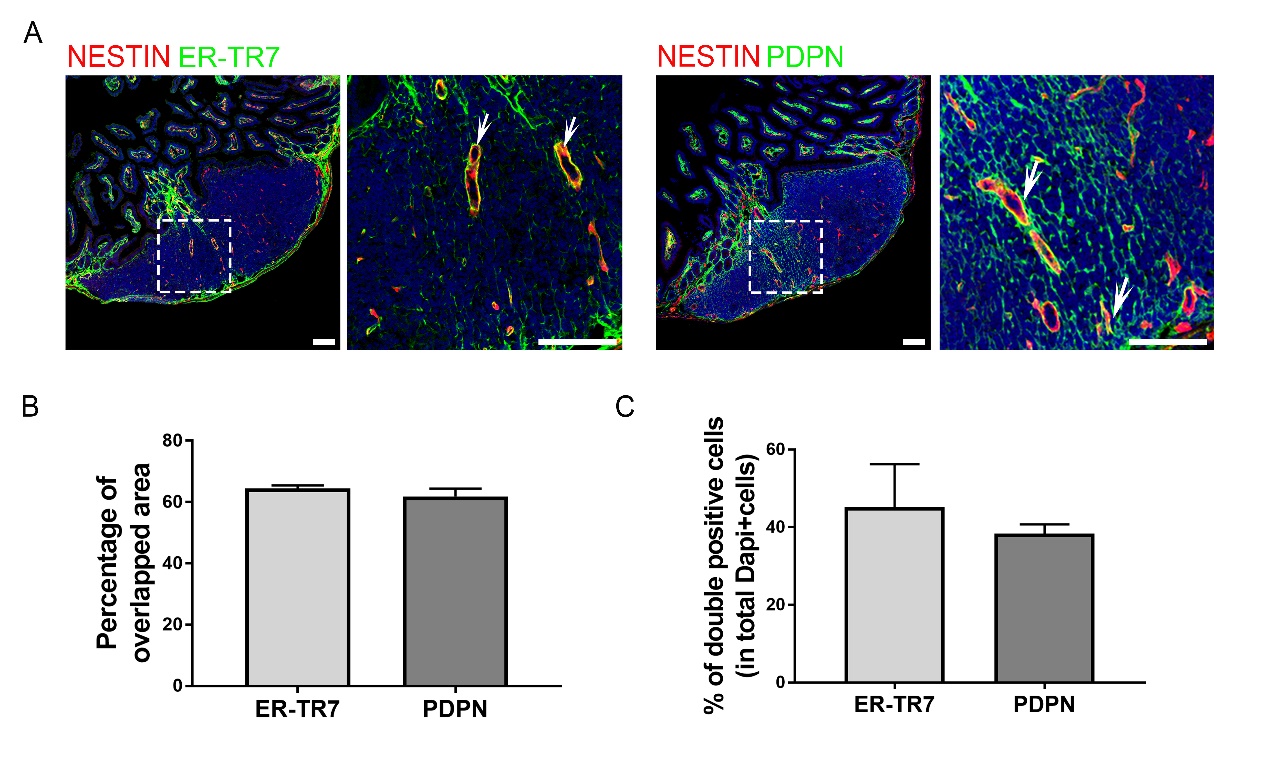
**

**Supplementary figure 1. Nestin expressing stromal cells are present in intestinal Peyer’s patches.**

**(A)** Representative images showed Primitive Peyer’s patches of Nestin-cre mice & Rosa26-td-Tomato(R26RtdT) reporter mice stained with DAPI (in blue) and stromal cell markers (in green) respectively, including ER-TR7, PDPN (indicated with an arrow). **(B)** Quantification of the overlapped percentage in identical area. **(C)** Quantification of the Nestin positive combined with specific stromal marker positive cells in identical area by ImageJ. Scale bars, 100μm.


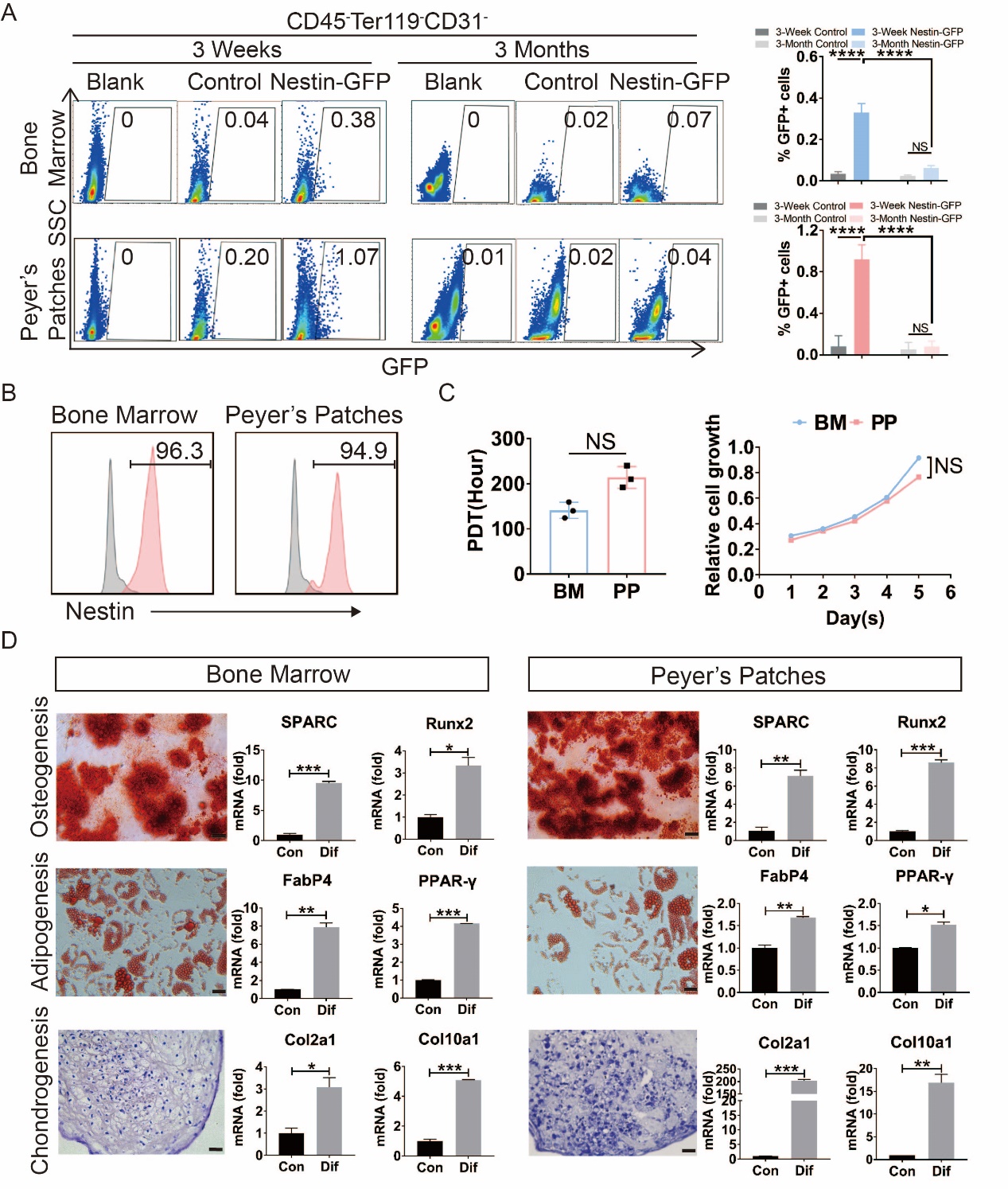


**Supplementary figure 2. Characterization of Nestin expressing cells from Bone marrow and Peyer’s patches of transgenic mice.**

**(A)** Bone marrow- and Peyer’s patches-derived CD45-Ter119-CD31-GFP+cells were detected by flow cytometry from 3-week-old and 3-month-old Nestin-GFP transgenic mice and non-transgenic C57BL/6 mice (as controls) respectively. Nestin+ cells in Bone marrow and Peyer’s patches were quantified into histogram respectively. **(B)** Nestin expressing on murine Bone marrow- and Peyer’s patches-derived Nestin+ cells respectively. **(C)** A Comparison of PDT between Bone marrow- and Peyer’s patches-derived Nestin+ cells and the proliferation rate was assessed using the CCK8 assay. **(D)** Representative images showing bone marrow– and Peyer’s patch–derived Nestin+ cells differentiating into osteocytes (alizarin red), adipocytes (oil red O) or chondrocytes (toluidine blue) in vitro; these findings were confirmed by qPCR analysis of the differentiation-associated genes, including SPARC and Runx2 (osteogenesis), FabP4 and PPAR-γ (adipogenesis), and Collagen II and Collagen X (chondrogenesis). The expression relatives to undifferentiated cells. Scale bars, 100μm. Data were shown as mean ± SD (n = 3). *P < 0.05, **P < 0.01, ***P < 0.001, ****P < 0.0001, NS, not significant; PDT, population doubling time; BM, bone marrow; PP, Peyer’s patches.

**
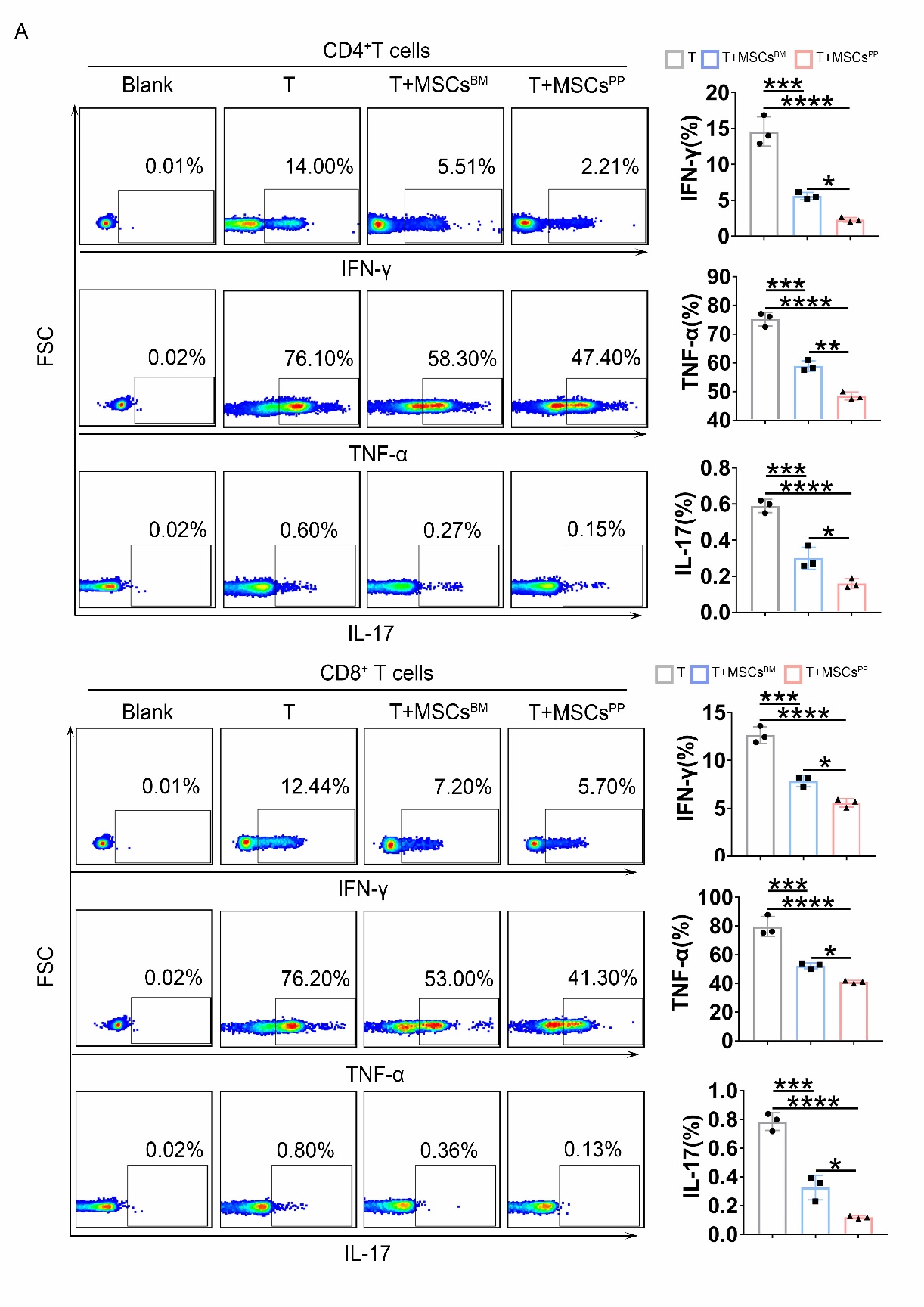
**

**Supplementary figure 3.** **MSCs^PP^ are more effective than MSCs^BM^ on suppressing the proinflammatory cytokines production of splenic T cells.**

**(A)**The secretion of IFN-γ, TNF-α, IL-17 in splenic CD4^+^T cells and CD8^+^T cells were analyzed after coculture with or without MSCs^BM/PP^. Data were shown as mean ± SD (n = 3). *P < 0.05, **P < 0.01, ***P < 0.001, ****P < 0.0001. IFN, interferon; TNF, tumor necrosis factor; IL, interleukin; MSCs^BM^, bone marrow-derived Nes+ MSCs; MSCs^PP^, Peyer’s patches-derived Nes+MSCs.


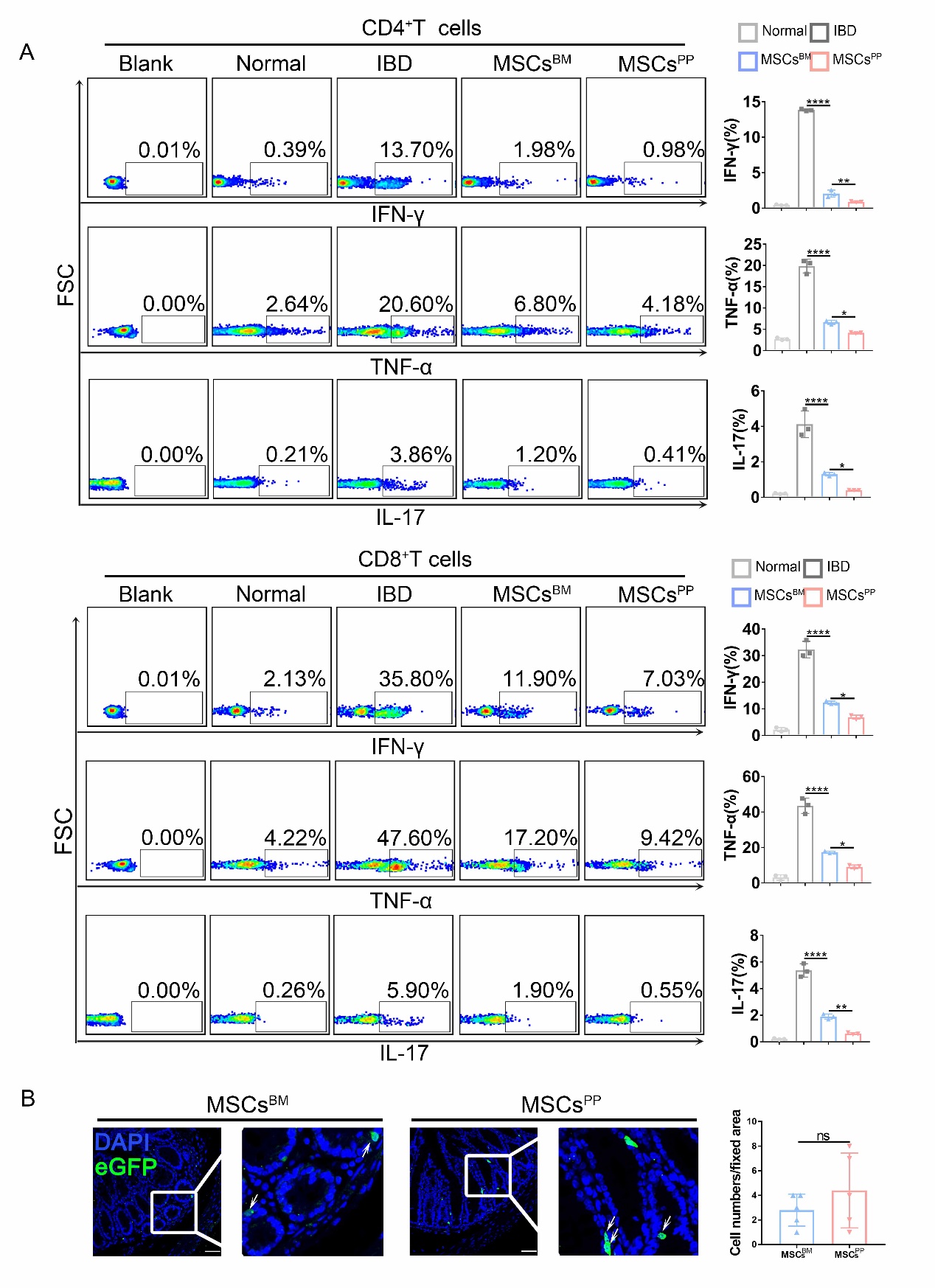


**Supplementary figure 4. MSCs^PP^ exhibit an enhanced therapeutic potential than MSCs^BM^ on inflammatory bowel disease.**

**(A)**The secretion of IFN-γ, TNF-α, IL-17 in CD4^+^T cells and CD8^+^T cells from Normal, IBD, MSCs^BM^ and MSCs^PP^ group were analyzed with flow cytometry. **(B)** The presence of eGFP-expressing MSCs in colons from MSCs^BM^ or MSCs^PP^ groups were examined by fluorescence microscopy and cell number was quantified into histogram. Scale bars, 50μm. Data were shown as mean ± SD (n = 3). *P < 0.05, **P < 0.01, ****P < 0.0001. NS, not significant. HE: hematoxylin and eosin; Normal, 0.9% NaCl control group; IBD, induced with TNBS model group; MSCs^BM^, model group treated with Nes+bmMSCs; MSCs^PP^, model group treated with Nes+ppMSCs.

**
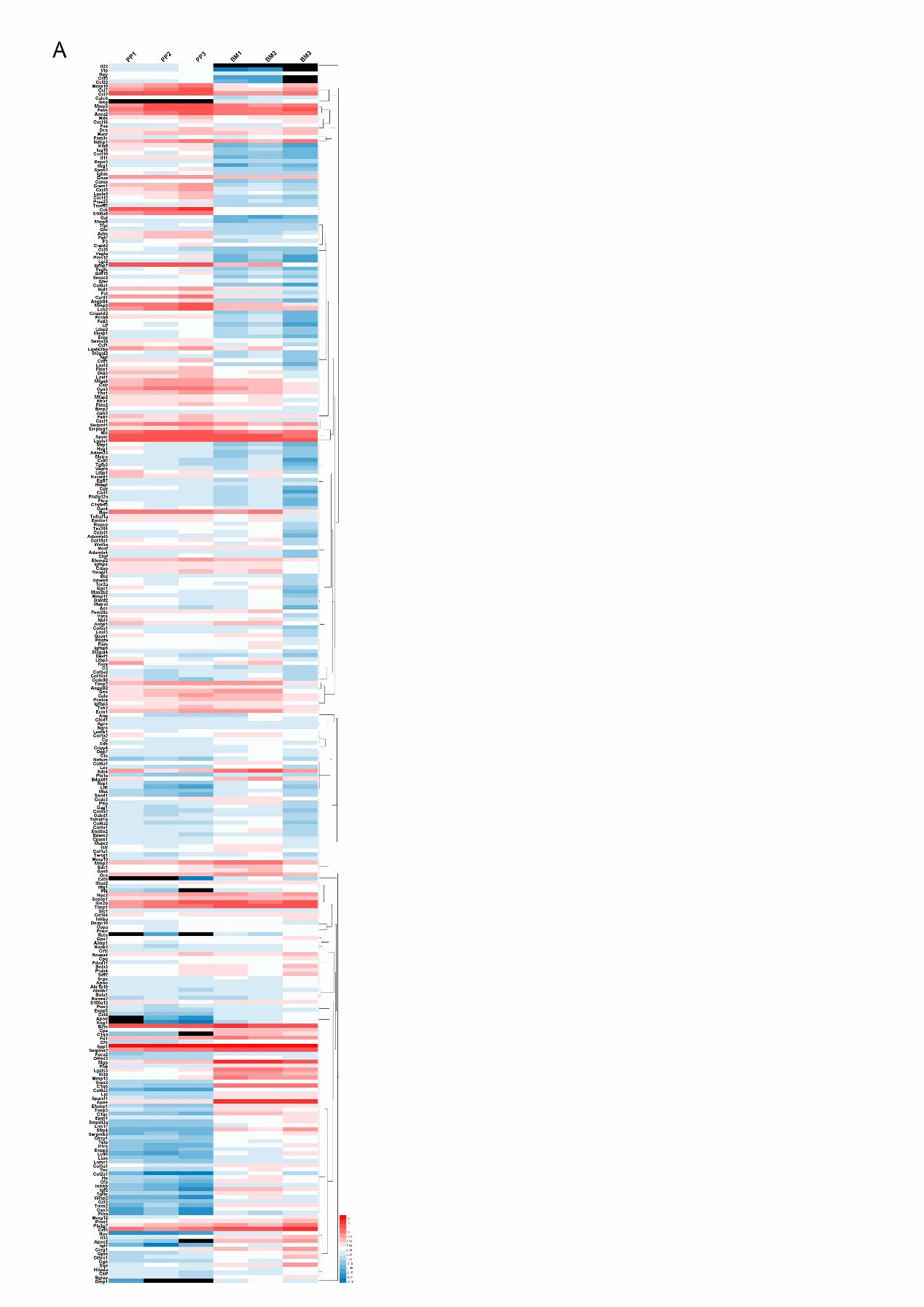
**

**Supplementary figure 5. 306 expressed genes of secretory proteins in MSCs^BM^ and MSCs^PP^.**

1. Heatmap plot showed 306 secretory protein genes that were expressing in MSCs^BM^ and MSCs^PP^.

**
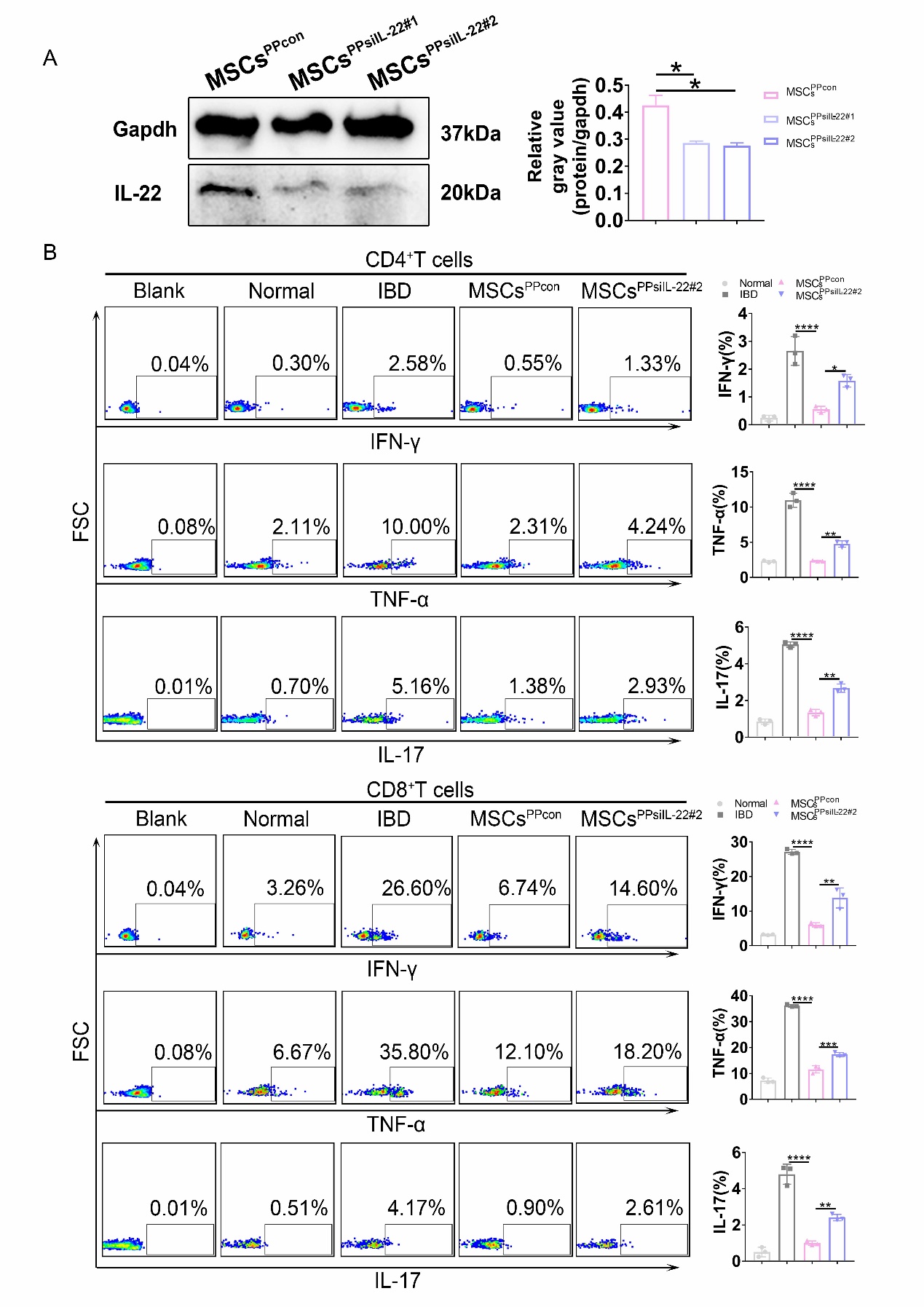
**

**Supplementary figure 6.** **MSCs^PP^ alleviate IBD partially through IL-22.**

**(A)** SiRNA-mediated down-regulation of IL-22 was assessed by western blot and the relative gray value was normalized to gapdh. **(B)** The secretion of IFN-γ, TNF-α, IL-17 in CD4^+^T cells and CD8^+^T cells from Normal, IBD, MSCs^PPcon^ and MSCs^PPsiIL22#2^ group were analyzed with flow cytometry. Data were shown as mean ± SD (n = 3). *P < 0.05, **P < 0.01, ***P < 0.001, ****P < 0.0001. Normal, 0.9% NaCl control group; IBD, induced with TNBS model group; MSCs^PPcon^, model group treated with control MSCs^PP^; MSCs^PPsiIL22#2^, model group treated with IL-22-knockdown MSCs^PP^.


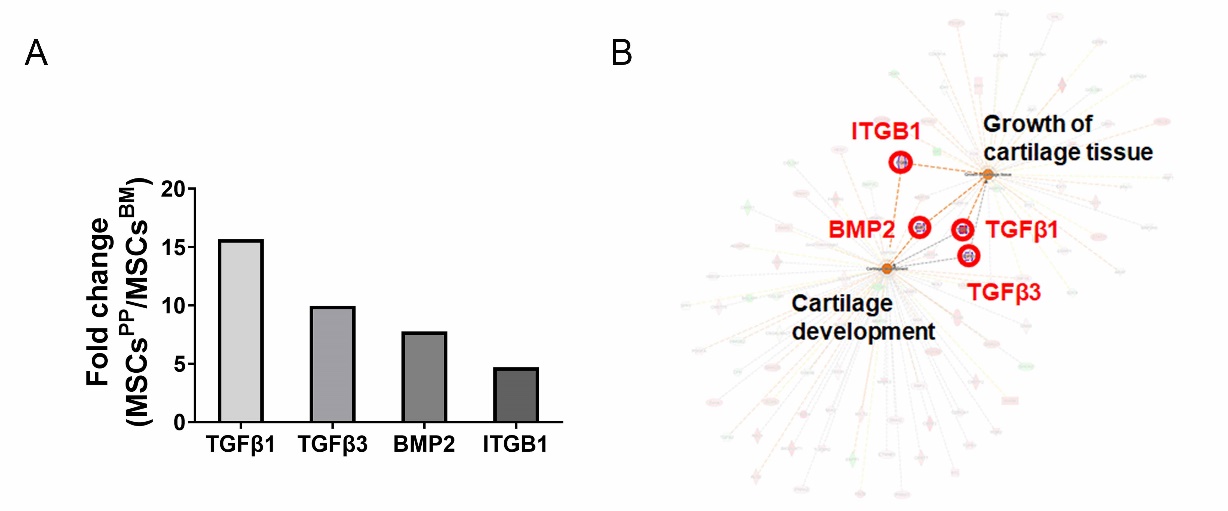


**Supplementary figure 7. Difference of cartilage associated genes between MSCs^PP^ and MSCs^BM^.**

**(A)** Fold changes of cartilage associated genes showed as MSCs^PP^/MSCs^BM^. **(B)** IPA analysis of cartilage growth and development genes significant higher on MSCs^PP^ than MSCs^BM^.
